# An optimized EfficientNetB0 framework with CLAHE-based preprocessing for accurate multi-class chest X-ray classification

**DOI:** 10.1038/s41598-026-42492-1

**Published:** 2026-03-28

**Authors:** Nagwa Yaseen Hegazy, Mohamed S. Sawah

**Affiliations:** 1 Information Systems Department, Faculty of Information Systems and Computer Science, October 6 University, Giza, 12585 Egypt; 2 Department of Data Science and Artificial Intelligence, Faculty of Information Technology, Ajloun National University, P.O.43, Ajloun, 26810 Jordan; 3 Department of Information Systems, Al-Alson Higher Institute, Cairo, Egypt

**Keywords:** Chest X-ray classification, EfficientNetB0, CLAHE preprocessing, Transfer learning, Thoracic pathology detection, Computational biology and bioinformatics, Diseases, Health care, Mathematics and computing, Medical research

## Abstract

Chest radiography remains an essential diagnostic tool for thoracic diseases, yet interpreting overlapping anatomical structures is particularly challenging when multiple pathologies co-occur a common clinical scenario often oversimplified in deep learning approaches. This study presents an optimized EfficientNetB0 framework designed explicitly for multi-label classification of chest X-rays using the NIH dataset, integrating CLAHE-based contrast enhancement, strategic class balancing, and a comparative transfer learning strategy that preserves the dataset’s inherent multi-label complexity. The proposed model achieved superior diagnostic performance with a macro-average AUC of 0.906 and recall of 0.824, outperforming DenseNet121 and MobileNetV2, and demonstrated strong per-class discrimination, especially for Pneumonia (AUC = 0.950) and Cardiomegaly (AUC = 0.946). These results confirm that the framework effectively balances learning capacity and generalization in a realistic multi-label clinical setting, offering a robust, interpretable solution suitable for computer-aided diagnosis where accurate detection of co-occurring thoracic pathologies is critical.

## Introduction

A medical imaging and diagnostic technique that is both economical and user-friendly is chest radiography (chest X-ray or CXR). The technique is the most frequently employed diagnostic instrument in medical practice and plays a critical role in the diagnosis of lung disease.

Chest X-rays are employed by radiologists who have received adequate training to identify a variety of maladies, including pneumonia, tuberculosis, interstitial lung disease, and early lung cancer^1^. The minimal cost and ease of operation of chest X-rays are among their greatest advantages. Modern digital radiography (DR) devices are exceedingly cost-effective, even in regions that are underdeveloped. As a result, chest radiographs are frequently employed to diagnose and detect lung diseases, including interstitial lung disease, tuberculosis, and pulmonary nodules. A significant quantity of information regarding a patient’s health is contained in chest radiography. Nevertheless, the physician consistently faces a significant obstacle in accurately interpreting the information. The interpretation is significantly complicated by the overlapping of the tissue structures in the thorax X-ray^2^.

The first attempt to establish a computer-aided detection system was in the 1960s^3^, and studies have shown that the detection accuracy for the chest disease is improved with a X-ray CAD system as an assistant. Many commercial products have been developed for the clinical applications, including CAD4 TB, Riverain, and Delft imaging systems^4^. However, because of the complexity of the chest X-rays, the automaticdetection of the diseases remains unresolved, and most of the existing CAD systems are aimed at the early detection of the lung cancer. A relatively small number of studies are devoted to the automatic detection of the other types of the pathologies^5^. The analysis and interpretation of medical imaging are being transformed by artificial intelligence (AI), which is revolutionizing radiology. Over the past decade, the AI industry in radiology has experienced exponential growth, with the U.S. Food and Drug Administration (FDA) approving over 100 companies and nearly 400 algorithms. The transformative potential of AI in addressing diagnostic challenges, such as identifying subtle abnormalities or characterizing irregular structures, is reflected in this growth, which frequently exceeds human capabilities^6^.

The field of medical imaging has experienced a significant increase in the use of computer-aided diagnosis (CAD) as a result of the recent surge in deep learning techniques. Commercial AI solutions for chest radiographs, which are designed using deep learning (DL) algorithms, have garnered attention and demonstrated exceptional performance in the detection of malignant pulmonary nodules, tuberculosis, and other abnormalities in experimental datasets, among the numerous applications of artificial intelligence (AI) in diagnostic imaging^7–9^.The AI solution’s diagnostic accuracy is superior to that of clinicians; however, experimentally collected datasets may have enriched disease prevalence, which may not be generalizable across disease domains. Consequently, in order to verify the AI solution’s efficacy in real-world clinical practice, cross-sectional studies should be implemented in carefully selected cohorts^10,11^.

AI algorithms have demonstrated high sensitivity and negative predictive value in diagnosing pulmonary embolism on CT pulmonary angiograms, complementing radiologists’ expertise^12^. For pulmonary nodule detection and classification, deep learning techniques have exhibited excellent performance, with one study reporting 94% sensitivity and 83% specificity for malignant nodule detection on chest radiographs^13^. AI applications extend to various lung conditions, including interstitial lung disease and chronic obstructive pulmonary disease^14^. Despite these advancements, challenges remain in implementing AI systems in clinical practice. The integration of AI is expected to enhance radiologists’ diagnostic confidence and efficiency rather than replace them^15^. Continued research and development in this field aim to improve early lung cancer detection and reduce associated morbidity and mortality.

In order to enhance the accuracy of chext X-ray classification, This study aims to develop an optimized deep learning framework for enhanced chest X-ray classification by integrating advanced preprocessing techniques and transfer learning with EfficientNetB0.

The primary objectives of this study are to: Systematically preprocess the publicly available chest X-ray dataset^16^ through greyscale conversion, CLAHE-based contrast enhancement, brightness adjustment, and strategic resizing (224 × 224), while addressing class imbalance via undersampling/oversampling. Implement rigorous data filtering by selecting target pathology classes and omitting multi-label images to ensure dataset integrity. Design and optimize a transfer learning pipeline using EfficientNetB0, comparing feature extraction versus fine-tuning approaches for classification efficacy. Comprehensively evaluate model performance through robust training/testing protocols and multi-metric validation to ensure clinical applicability.

The primary contributions of this work are:

1. We introduce a systematic, reproducible, and clinically-informed preprocessing pipeline specifically designed for chest X-ray images. This pipeline integrates grayscale preservation, optimized CLAHE-based contrast enhancement (clipLimit = 2.0, tileGridSize = 8 × 8), brightness adjustment, and standardized resizing. The parameters are selected based on radiological best practices to enhance subtle thoracic structures and standardize inputs for deep learning, while remaining adaptable to other medical imaging modalities.
2. We propose a rigorous data balancing strategy that preserves the NIH dataset’s inherent multi-label complexity, addressing severe class imbalance without discarding clinically relevant multi-label samples. Our per-class strategy employs strategic oversampling and undersampling, ensuring robust learning in a setting that reflects the real-world co-occurrence of thoracic pathologies.
3. We design and validate an enhanced EfficientNetB0 framework optimized for multi-label thoracic classification, integrating a custom Squeeze-Excitation attention block for improved feature recalibration and employing Focal Loss to handle class imbalance and label noise. The framework implements a two-phase transfer learning strategy (feature extraction followed by full fine-tuning) evaluated through comprehensive metrics, providing empirical guidance on optimal training protocols for multi-label medical image analysis.

The remainder of this paper is organized as follows: section “Related work” (Related Work) critically examines existing deep learning approaches for chest X-ray classification, emphasizing gaps in integrated preprocessing pipelines and transfer learning strategies. Section  “Proposed methodology” (Methodology) details the data preprocessing workflow including greyscale conversion, CLAHE-based contrast enhancement, brightness adjustment, resizing (224 × 224), and class-balancing (undersampling/oversampling) followed by data filtering protocols for class selection and multi-label omission, and the EfficientNetB0 implementation for feature extraction and fine-tuning. Section “Results and discussion” (Results and Discussions) presents quantitative performance analysis across training, validation, and testing phases. Section “Conclusion” (Conclusion) synthesizes the contributions of the optimized framework, discusses clinical implications, and proposes future research directions for real-world deployment. Finally, Sect. “Limitations and future directions” describes the limitation and future directions.

## Related work

In this literature section, we review key research on the application of deep learning to chest X-ray analysis. We highlight various approaches, models, and outcomes, which are also briefly summarized in Table 1. This review helps to identify gaps and opportunities for further investigation in our study.

A deep learning framework tailored for the multi-class diagnosis of lung diseases is ntroduced in^17^, including fibrosis, opacity, tuberculosis, viral pneumonia, COVID-19 pneumonia, and normal cases, using chest X-ray images. The framework leverages a custom convolutional neural network (CNN) architecture designed to extract discriminative features effectively. The study addresses challenges like dataset imbalance by employing data augmentation techniques. Extensive experiments demonstrate superior performance, achieving an accuracy of 98.88% and strong performance metrics such as an F1-score of 0.9887 and an AUC of 0.9939. This study highlights the potential of deep learning in enhancing diagnostic accuracy and efficiency in medical imaging, contributing significantly to automated lung disease detection and management.

The study in^18^ proposed a biphasic majority voting-based system for the automated diagnosis of COVID-19, normal, and pneumonia cases using chest X-ray images. Their method utilized six classifiers, selecting the five best-performing ones in two phases. Features were extracted with the Bag of Features method and classifiers like KNN, Linear Discriminant, Logistic Regression, and SVM. The approach achieved high accuracy rates of 99.86% (Phase-1) and 99.28% (Phase-2), with an overall accuracy of 99.63%. Notably, the system also demonstrated excellent performance across metrics like specificity, precision, recall, and F1-score. The study emphasized the importance of usability, integrating a graphical user interface (GUI) for accessibility by non-experts. Their results showed superior performance compared to similar models, highlighting the reliability of the biphasic majority voting technique.

A high-precision multiclass classification model is proposed in^19^, MobileLungNetV2, for diagnosing lung diseases using the ChestX-ray14 dataset. The model, fine-tuned from MobileNetV2, achieved 96.97% classification accuracy, outperforming other models like InceptionV3 and VGG19. Image pre-processing techniques such as CLAHE and Gaussian filtering were used to improve data quality. The model demonstrated high precision (96.71%), recall (96.83%), and specificity (99.78%), and utilized Grad-CAM for visualizing disease detection areas. The study highlights the effectiveness of MobileNetV2 in automated lung disease classification^19^.

The study in^20^ proposed two deep learning approaches for classifying and localizing lung abnormalities, including COVID-19, on chest X-rays. The study utilized multi-classification and object detection models trained on a large chest X-ray dataset. By combining multiple object detection models, the approach outperformed single object models in both classification and localization tasks. The method achieved promising results and has the potential to assist radiologists in diagnosing chest X-ray abnormalities more accurately and efficiently, improving patient outcomes and reducing healthcare system burdens.

The CXR-LT challenge is presented in^21^, focusing on the long-tailed, multi-label disease classification problem in chest X-ray (CXR) imaging. Medical image recognition, particularly for chest radiography, is often long-tailed, with a few common findings overshadowed by many rare conditions. The challenge addressed both label imbalance and co-occurrence of multiple diseases in patients. They released a large-scale dataset of over 350,000 CXRs with 26 clinical findings, emphasizing the need for specialized techniques to tackle these challenges. The study also discussed top-performing solutions and proposed using vision-language foundation models for few and zero-shot disease classification, offering practical recommendations for future research in this area.

An optimized ensemble framework for multi-label classification on long-tailed chest X-ray data is introduced in^22^, addressing the challenge of diagnosing multiple diseases from chest X-ray images. The paper highlights the complexities of multi-label classification in medical imaging, where patients often present with multiple overlapping diseases. This challenge is compounded by the long-tailed distribution of diseases, where common conditions are overshadowed by rare ones, leading to biased predictions. The authors focus on the MIMIC-CXR-LT dataset, which is designed to address these issues in multi-label long-tailed classification. Their optimized ensemble approach, which involves experimentation with architecture design and data augmentation, improved classification performance on imbalanced medical images. The proposed framework ranked highly in the CXR-LT competition, demonstrating its effectiveness in tackling the long-tailed distribution and multi-label classification problems in medical imaging.

An Artificial Intelligence (AI)-based classification system is proposed in^23^ to differentiate between COVID-19 and other infectious diseases using chest X-ray images. The study addresses the global health crisis caused by the COVID-19 pandemic, focusing on the need for faster diagnostic methods to supplement or replace RT-PCR tests. The authors utilized publicly available PA chest X-ray images of adult COVID-19 patients to train deep learning models for rapid screening. To enhance the dataset and improve model generalization, they performed 25 types of image augmentation. The models were trained using a transfer learning approach, and the combination of two best-performing models yielded the highest prediction accuracy for categories including normal, COVID-19, non-COVID-19 pneumonia, and tuberculosis. The results suggest that their AI-based method outperforms previous models in terms of efficiency and accuracy, offering promising advancements for biomedical imaging in COVID-19 diagnostics.

The study in^24^ proposed a multichannel deep learning approach for detecting lung diseases from chest X-ray images. The model uses EfficientNetB0, EfficientNetB1, and EfficientNetB2 pretrained models to extract features, which are then fused together and passed through non-linear fully connected layers. The fused features are further processed using a stacked ensemble learning classifier, which combines random forest, support vector machine (SVM), and logistic regression for lung disease detection. The method was tested on several lung diseases, including pneumonia, tuberculosis (TB), and COVID-19, achieving impressive performance with 98% detection accuracy for pediatric pneumonia, 99% for TB, and 98% for COVID-19. The proposed method outperformed similar techniques, demonstrating robust performance on unseen data and offering potential as a reliable tool for point-of-care diagnosis by radiologists. The feature optimization was also visualized using t-SNE for further validation of the model’s efficiency.

The author in^25^ proposed a deep learning method using transfer learning to classify lung diseases from chest X-ray (CXR) images. Their method employs an end-to-end learning approach where raw CXR images are directly inputted into the EfficientNet v2-M model to extract meaningful features for disease classification. The study was tested on two datasets: the U.S. National Institutes of Health (NIH) dataset and the Cheonan Soonchunhyang University Hospital (SCH) dataset. For the NIH dataset, which included normal, pneumonia, and pneumothorax classes, the method achieved a validation accuracy of 82.15%, with 81.40% sensitivity and 91.65% specificity. For the SCH dataset, which added tuberculosis as a fourth class, the method achieved a validation accuracy of 82.20% and 94.48% specificity. The study demonstrates the potential of transfer learning to enhance the efficiency and accuracy of computer-aided diagnostic systems (CADs) for lung disease classification.

A multi-classification deep learning model named CDC-Net id introduced in^26^ for detecting COVID-19, lung cancer (LC), pneumothorax, tuberculosis (TB), and pneumonia from chest X-ray images. The model incorporates residual networks and dilated convolution techniques, aiming to improve early diagnosis of these diseases, especially considering their similar symptoms that can mislead clinical professionals. The study used publicly available benchmark datasets to train and test the model, which outperformed several pre-trained CNN models, including VGG-19, ResNet-50, and Inception v3. The CDC-Net achieved an impressive accuracy of 99.39%, recall of 98.13%, and precision of 99.42%, with an AUC of 0.9953 for multi-disease classification. In comparison, the pre-trained models achieved lower accuracies: 95.61%, 96.15%, and 95.16%, respectively. Statistical tests (McNemar’s and ANOVA) confirmed the robustness of CDC-Net, suggesting its high potential for reliable and accurate chest disease diagnosis.

The study in^27^ proposed a deep learning architecture for multi-class lung disease classification using chest X-ray (CXR) images. This model aimed to classify several lung conditions, including COVID-19, pneumonia, lung cancer, tuberculosis (TB), and lung opacity, which often share similar symptoms, making accurate and early diagnosis challenging. The dataset consisted of 3615 COVID-19 images, 6012 lung opacity, 5870 pneumonia, 20,000 lung cancer, 1400 TB, and 10,192 normal images. The model employed the pre-trained VGG19 architecture, followed by three blocks of convolutional neural networks (CNN) for feature extraction and a fully connected network for classification. The proposed approach achieved remarkable performance with 96.48% accuracy, 93.75% recall, 97.56% precision, 95.62% F1 score, and an AUC of 99.82%, outperforming existing methods. This high performance could significantly assist healthcare practitioners by enabling faster and more accurate diagnoses.

**Table 1:** Show the comparison comparison of lung disease classification models using chest X-ray images.

Author name	Dataset	Used algorithm	Dataset size	Accuracy	Advantages	Disadvantages
Sanida et al.^26^	Chest X-ray images (multi-class)	Custom CNN with VGG19 architecture	21,165 X-ray images	98.88%	High diagnostic accuracy with advanced feature extraction and data augmentation	Dataset imbalance required augmentation; computational cost due to architectural complexity
Sunnetci and Alkan^26^	Chest X-ray images (binary & multi-class)	Bag of Features + Multiple Classifiers	~ 10,000 images	99.63%	High accuracy using a biphasic majority voting method, user-friendly GUI	Limited generalization to unseen data; reliance on predefined feature extraction methods
Shamrat et al.^26^	Chest X-ray images	Fine-tuned MobileNetV2 (MobileLungNetV2)	112,120 X-ray images (ChestX-ray14)	96.97%	High classification accuracy; effective use of CLAHE and Gaussian filters for pre-processing; Grad-CAM for visual explanations	Computational complexity; requires high computational power due to fine-tuning and large dataset size
Elhanashi et al.^26^	Chest X-ray images	Deep learning (multi-classification and ensemble object detection)	Large dataset (not specified in detail)	Promising results (accuracy not provided)	Enables both classification and localization; employs ensemble models for improved detection; applicable to multiple diseases, including COVID-19	Dataset size and computational requirements not detailed; results compared qualitatively, lacking numerical specificity
Holste et al.^26^	Chest X-ray images	Long-tailed, multi-label classification models	350,000 CXRs	Not specified	long-tailed and multi-label challenges; includes 26 clinical findings; large-scale benchmark dataset released for research	Accuracy metrics not detailed; solutions proposed but lack performance quantification for individual models
Jeong et al.^26^	Chest X-ray images	Optimized ensemble framework for multi-label, long-tailed classification	MIMIC-CXR-LT dataset (size not specified)	Not specified	Addresses multi-label and long-tailed distribution; employs data augmentation and optimized ensemble models; ranks high in the CXR-LT competition	Specific accuracy metrics not provided; focused on competition performance, limiting insights for general applications
Sharma et al.^26^	Chest X-ray images	Transfer learning-based deep learning	286 images (rotated through 120° or 140° with 25 augmentations)	Not explicitly stated; combination of models displayed the highest prediction accuracy	Efficient classification of COVID-19 and other infectious diseases; utilizes transfer learning and image augmentation	Small initial dataset size; dependent on image augmentation and transfer learning to generalize
Ravi et al.^26^	Chest X-ray images	Multichannel EfficientNet-based stacking ensemble	Not explicitly stated	98% for pediatric pneumonia, 99% for TB, 98% for COVID-19	Robust and generalizable; employs multichannel models (EfficientNetB0, B1, B2); stacked ensemble improves performance	Lacks dataset size details; computationally intensive due to multichannel and stacked ensemble architecture
Kim et al.^26^	Chest X-ray images	Transfer learning with EfficientNet v2-M	NIH dataset (unspecified size) and SCH dataset (unspecified size)	82.15% (NIH), 82.20% (SCH); per-class accuracy: 63.60%-89.90%	End-to-end learning approach; good sensitivity (81.40%) and specificity (up to 94.48%)	Lower testing accuracy for certain classes; lack of dataset size details; variable performance across classes
Malik et al.^26^	Chest X-rays of COVID-19, pneumothorax, pneumonia, lung cancer, and tuberculosis	Publicly available benchmark datasets	Convolutional Neural Network (CDC Net) with residual network and dilated convolution	99.39%	Highly accurate for multi-class classification Incorporates residual networks and dilated convolutions Outperforms pre-trained models (e.g., VGG-19, ResNet-50, Inception v3)	Relies on publicly available datasets, which might not represent real-world diversity Potential limitations in generalization due to dataset specifics
Alshmrani et al.^26^	Chest X-rays for COVID-19, Pneumonia, Lung Cancer, Tuberculosis, Lung Opacity, and Normal	VGG19 + Convolutional Neural Network (CNN)	3615 COVID-19, 6012 Lung Opacity, 5870 Pneumonia, 20,000 Lung Cancer, 1400 Tuberculosis, 10,192 Normal (total ~ 47,089 images)	96.48%	Combines pre-trained VGG19 with additional CNN layers High precision and AUC (99.82%) Superior performance in multi-class classification	Imbalanced dataset sizes may impact generalization Performance depends heavily on the quality of pre-trained model

## Proposed methodology

### Overview

**Fig. 1:**
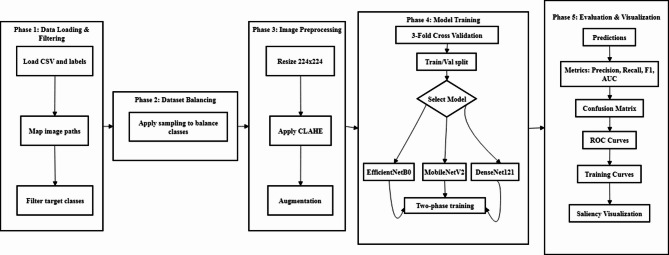
Proposed methodology.

The proposed framework was implemented as shown in Fig. 1, following a structured pipeline comprising data preparation, preprocessing, model architecture design, training configuration, and comprehensive evaluation. The methodology preserves the inherent multi-label nature of chest X-ray classification to maintain clinical relevance.

### Dataset description and preparation

We utilized the NIH ChestX-ray dataset released by the NIH Clinical Center, comprising 112,120 frontal-view chest radiographs from 30,805 unique patients. We used the official 2017 release (including the accompanying metadata file Data_Entry_2017.csv) with labels mined from the associated radiology reports using natural language processing (NLP), as originally described in^29^. The dataset was downloaded from the official NIH repository^16^. The dataset is publicly available and de-identified. We used the data under the dataset’s stated data-use and attribution terms and did not attempt to re-identify any individuals. For clinical relevance, we focused on five key thoracic pathologies: No Finding, Pneumonia, Pneumothorax, Effusion, and Cardiomegaly. The dataset was structured for multi-label classification where each image can have zero, one, or multiple positive labels simultaneously.

*Class balancing strategy*:

Medical datasets typically exhibit severe class imbalance. To address this, we implemented a per-class balancing approach with the following target sample counts based on clinical prevalence and learning requirements. For each class, we applied either oversampling with replacement (for underrepresented classes) or undersampling without replacement (for overrepresented classes). This approach maintained the dataset’s multi-label nature while ensuring balanced representation during training.

### Preprocessing pipeline

Our preprocessing pipeline is designed to enhance radiographic features while maintaining clinical relevance:

1. Grayscale Preservation: Images were maintained in grayscale format, consistent with diagnostic imaging standards.
2. CLAHE Enhancement: We applied Contrast Limited Adaptive Histogram Equalization (CLAHE) with clinically optimized parameters (clipLimit = 2.0, tileGridSize = ( 8,8)). This locally adaptive contrast enhancement highlights subtle radiodensity variations crucial for pathology detection while avoiding over-amplification of noise.
3. Standardized Resizing: All images were resized to 224 × 224 pixels to ensure compatibility with the EfficientNetB0 architecture while preserving aspect ratio through proper interpolation.
4. Brightness Adjustment: A uniform brightness adjustment was applied to improve visibility of anatomical structures.
5. Normalization: Pixel values were scaled to the [0, 255] range for compatibility with EfficientNet’s preprocessing requirements.

#### Data Augmentation:

To prevent overfitting and improve generalization, we applied the following real-time augmentations during training.

- Horizontal flipping (50% probability).
- Random rotation within ± 7 degrees.
- Random brightness (± 12%) and contrast (± 15%) adjustments.
- Small-scale zoom variations (± 10%).

The preprocessing pipeline is not dataset-specific and can be adapted to other medical imaging modalities with similar characteristics (grayscale images, class imbalance, multi-label annotations).

### Model Architecture

The core of our framework is an enhanced EfficientNetB0 architecture optimized for multi-label chest X-ray classification:

#### Backbone:

EfficientNetB0 pretrained on ImageNet served as the feature extractor, providing a robust foundation for medical image analysis while maintaining computational efficiency.

*Architectural Enhancements*:

1. Squeeze-Excitation Attention Block: We incorporated a custom Squeeze-Excitation (SE) block after the base model to enhance discriminative feature learning. This attention mechanism adaptively recalibrates channel-wise feature responses, improving sensitivity to subtle pathological features.
2. Multi-Label Output Layer: The model employs five independent sigmoid activation units, each corresponding to one pathology class. This design enables true multi-label classification by allowing simultaneous prediction of multiple conditions with independent probability estimates.
3. Regularization Components:
  Global Average Pooling to reduce parameter count while preserving spatial hierarchy.
  Batch Normalization layers to stabilize training and accelerate convergence.
  Dropout (0.4 rate) to prevent co-adaptation of neurons.
  L2 regularization to constrain model complexity.

#### Loss Function:

We employed Focal Loss (γ = 2.0, α = 0.25). This advanced loss function down-weights well-classified examples and focuses training on challenging, misclassified cases, which is particularly effective for handling class imbalance and label noise.

### Training Strategy

Our training protocol employed a sophisticated two-phase transfer learning approach:

*Phase 1 (Feature Extraction)*:

- Epochs: 5 (or until convergence).
- Learning Rate: 1e−4.
- Configuration: EfficientNetB0 backbone frozen, only classification head trainable.
- Objective: Learn dataset-specific feature representations while leveraging ImageNet knowledge.

*Phase 2 (Fine-Tuning)*:

- Epochs: Up to 20 (total 25 epochs maximum).
- Learning Rate: 5e−5 (reduced for stable fine-tuning).
- Configuration: Entire model unfrozen for end-to-end optimization.
- Objective: Refine both feature extraction and classification for the specific thoracic pathology domain.

**Training Optimization**:

- Optimizer: Adam with adaptive learning rate scheduling.
- Batch Size: 16 (balanced between computational efficiency and gradient stability).
- Early Stopping: Monitored validation loss with patience of 5–6 epochs.
- Model Checkpointing: Saved best-performing weights based on validation metrics.
- Learning Rate Reduction: Activated when validation loss plateaued (factor = 0.3, patience = 2–3).

#### Cross-Validation:

We employed rigorous threefold cross-validation to ensure robust performance estimation and minimize bias from data partitioning.

### Evaluation framework

Comprehensive evaluation was conducted using multiple metrics^28^ appropriate for multi-label classification:

- AUC (Area Under ROC Curve): Primary measure of discriminative ability.
- Precision, Recall, F1-Score: Balanced assessment of classification performance.
- Confusion Matrices: Visualization of classification patterns and error types.
- Standard Deviation: Measure of performance stability across folds.

The evaluation framework ensures clinically relevant assessment, with particular emphasis on sensitivity (recall) for critical conditions like pneumothorax and pneumonia, where false negatives have significant clinical consequences.

### Implementation details

The complete pipeline was implemented in Python using TensorFlow/Keras, with all preprocessing, augmentation, training, and evaluation steps integrated into a reproducible workflow. The code is structured to facilitate adaptation to other medical imaging tasks and datasets, with comprehensive configuration options for key hyperparameters.

## Results and discussion

The threefold cross-validation results for DenseNet121 are presented in Table 2. The model achieved a consistent average validation accuracy of 89% across all folds, with minimal variability as indicated by low standard deviations in performance metrics (e.g., F1-score standard deviations ranged from 0.0046 to 0.0175). Class-wise analysis revealed that Cardiomegaly achieved the highest average AUC (0.94), while Pneumothorax exhibited the lowest average F1-score (0.64). The stability across folds suggests DenseNet121 provides reliable but moderate performance for thoracic pathology classification.

**Table 2:** Show the results of DensNet121.

Fold	Class	Precision	Recall	F1-score	ROC-AUC
Fold(1)	No Finding	0.71	0.73	0.72	0.86
	Pneumonia	0.62	0.86	0.72	0.93
	Pneumothorax	0.51	0.81	0.63	0.89
	Effusion	0.66	0.81	0.73	0.87
	Cardiomegaly	0.67	0.83	0.74	0.94
Fold(1)—validation accuracy	0.89	–	–	–	–
Fold(2)	No Finding	0.70	0.72	0.71	0.86
	Pneumonia	0.60	0.85	0.70	0.93
	Pneumothorax	0.57	0.75	0.65	0.89
	Effusion	0.70	0.77	0.73	0.86
	Cardiomegaly	0.63	0.87	0.73	0.94
Fold(2)—validation accuracy	0.89	–	–	–	–
Fold(3)	No Finding	0.69	0.75	0.72	0.87
	Pneumonia	0.65	0.84	0.73	0.93
	Pneumothorax	0.53	0.81	0.64	0.89
	Effusion	0.69	0.77	0.73	0.87
	Cardiomegaly	0.61	0.83	0.70	0.93
Fold(3)—validation accuracy	0.89	–	–	–	–
Class average	No Finding	0.70	0.73	0.72	0.86
	Pneumonia	0.62	0.85	0.72	0.93
	Pneumothorax	0.54	0.79	0.64	0.89
	Effusion	0.68	0.78	0.73	0.87
	Cardiomegaly	0.64	0.84	0.72	0.94
Class standard deviation	No Finding	0.0087	0.0133	0.0046	0.0018
	Pneumonia	0.0218	0.0081	0.0132	0.0017
	Pneumothorax	0.0239	0.0251	0.0095	0.0032
	Effusion	0.0177	0.0173	0.0028	0.0042
	Cardiomegaly	0.0249	0.0180	0.0175	0.0060
Average validation accuracy	0.89	–	–	–	–

Table 3 summarizes MobileNetV2’s performance through threefold cross-validation, yielding an average validation accuracy of 88%. The model demonstrated particularly strong performance for Pneumonia, achieving the highest average F1-score (0.83) and AUC (0.95) among all classes. However, it showed relative weakness in classifying Pneumothorax, with the lowest average F1-score (0.61) and precision (0.50). The moderate standard deviations across folds (e.g., F1-score SDs: 0.0055–0.0193) indicate reasonable consistency, though slightly higher variability compared to DenseNet121.

**Table 3:** Show the results of MobileNetV2.

Fold	Class	Precision	Recall	F1-score	ROC-AUC
Fold(1)	No Finding	0.69	0.75	0.72	0.86
	Pneumonia	0.81	0.87	0.84	0.95
	Pneumothorax	0.48	0.81	0.60	0.88
	Effusion	0.70	0.78	0.74	0.87
	Cardiomegaly	0.60	0.85	0.71	0.94
Fold(1)—validation accuracy	0.88	–	–	–	–
Fold(2)	No Finding	0.63	0.81	0.71	0.86
	Pneumonia	0.81	0.87	0.84	0.95
	Pneumothorax	0.50	0.77	0.61	0.87
	Effusion	0.65	0.81	0.73	0.86
	Cardiomegaly	0.62	0.86	0.72	0.94
Fold(2)—validation accuracy	0.88	–	–	–	–
Fold(3)	No Finding	0.66	0.78	0.71	0.86
	Pneumonia	0.79	0.85	0.82	0.95
	Pneumothorax	0.52	0.77	0.62	0.88
	Effusion	0.69	0.77	0.72	0.87
	Cardiomegaly	0.65	0.84	0.74	0.94
Fold(3)—validation accuracy	0.88	–	–	–	–
Class average	No Finding	0.66	0.78	0.71	0.87
	Pneumonia	0.80	0.87	0.83	0.95
	Pneumothorax	0.50	0.78	0.61	0.87
	Effusion	0.68	0.79	0.73	0.87
	Cardiomegaly	0.63	0.85	0.72	0.94
Class standard deviation	No Finding	0.0271	0.0247	0.0055	0.0015
	Pneumonia	0.0065	0.0083	0.0073	0.0023
	Pneumothorax	0.0170	0.0180	0.0078	0.0031
	Effusion	0.0206	0.0205	0.0067	0.0037
	Cardiomegaly	0.0213	0.0072	0.0116	0.0019
Average validation accuracy	0.88	–	–	–	–

Table 4 show EfficientNetB0’s cross-validation results, with an average validation accuracy of 89%. The model achieved the highest average AUC values across multiple classes, particularly for Cardiomegaly (0.95) and Pneumonia (0.94). While Pneumothorax classification remained challenging (average F1-score: 0.63), EfficientNetB0 showed improved recall for this class (0.81) compared to other architectures. The model exhibited moderate variability across folds, with standard deviations for F1-scores ranging from 0.0041 to 0.0254.

**Table 4:** Show the results of EfficientNetB0.

Fold	Class	Precision	Recall	F1-Score	ROC-AUC
Fold(1)	No Finding	0.71	0.75	0.73	0.87
	Pneumonia	0.72	0.86	0.79	0.95
	Pneumothorax	0.48	0.83	0.61	0.90
	Effusion	0.69	0.82	0.75	0.88
	Cardiomegaly	0.67	0.85	0.75	0.95
Fold(1)—validation accuracy	0.89	–	–	–	–
Fold(2)	No Finding	0.68	0.76	0.72	0.87
	Pneumonia	0.70	0.89	0.78	0.95
	Pneumothorax	0.52	0.79	0.63	0.89
	Effusion	0.70	0.78	0.74	0.87
	Cardiomegaly	0.59	0.89	0.71	0.95
Fold(2)—validation accuracy	0.88	–	–	–	–
Fold(3)	No Finding	0.68	0.76	0.72	0.87
	Pneumonia	0.65	0.84	0.73	0.93
	Pneumothorax	0.52	0.81	0.63	0.89
	Effusion	0.66	0.82	0.73	0.87
	Cardiomegaly	0.59	0.88	0.71	0.94
Fold(3)—validation accuracy	0.89	–	–	–	–
Class average	No Finding	0.69	0.76	0.72	0.87
	Pneumonia	0.69	0.87	0.77	0.94
	Pneumothorax	0.51	0.81	0.63	0.89
	Effusion	0.69	0.81	0.74	0.88
	Cardiomegaly	0.62	0.87	0.72	0.95
Class standard deviation	No Finding	0.0101	0.0031	0.0041	0.0030
	Pneumonia	0.0310	0.0224	0.0254	0.0101
	Pneumothorax	0.0188	0.0179	0.0095	0.0046
	Effusion	0.0159	0.0184	0.0070	0.0049
	Cardiomegaly	0.0355	0.0133	0.0193	0.0049
Average validation accuracy	0.89	–	–	–	–

Table 5 provides a comprehensive comparison of the three architectures’ performance metrics averaged across threefold cross-validation. DenseNet121 and EfficientNetB0 both achieved the highest average validation accuracy (89.00%), while MobileNetV2 showed slightly lower performance (88.00%). MobileNetV2 exhibited the highest macro-average precision (0.654) and F1-score (0.720), indicating strong classification consistency. However, EfficientNetB0 demonstrated superior sensitivity with the highest macro-average recall (0.824) and discriminative ability with the highest macro-average AUC (0.906), suggesting better identification of positive cases across all pathologies. DenseNet121 showed the most stable performance with the lowest average standard deviation in F1-scores (0.0115), indicating minimal variability across folds. This comparative analysis reveals that while MobileNetV2 offers strong precision-based performance, EfficientNetB0 provides a better balance between sensitivity (recall) and discriminative power (AUC), making it particularly suitable for clinical applications where false negatives carry significant consequences.

**Table 5:** Comparative Analysis of model performance across threefold cross-validation.

Model	Avg. val. accuracy (%)	Macro avg. precision	Macro avg. recall	Macro avg. F1-score	Macro avg. AUC	Avg. std dev (F1)
DenseNet121	89.00	0.636	0.798	0.706	0.898	0.0115
MobileNetV2	88.00	0.654	0.814	0.720	0.900	0.0118
EfficientNetB0	89.00	0.640	0.824	0.716	0.906	0.0131

The comprehensive analysis reveals EfficientNetB0 as the optimal architecture for thoracic pathology classification based on three key factors: (1) It achieved the highest discriminative ability with a macro-average AUC of 0.906, indicating superior separation between pathological and normal cases; (2) It demonstrated the best sensitivity with a macro-average recall of 0.824, crucial for minimizing false negatives in critical conditions like pneumonia and pneumothorax; (3) While MobileNetV2 showed marginally better precision (0.654 vs. 0.640) and F1-score (0.720 vs. 0.716), EfficientNetB0’s superior AUC and recall metrics better align with clinical priorities in thoracic screening.

Among the threefolds of EfficientNetB0, Fold 1 emerges as the most representative configuration for generating final evaluation figures based on the following quantitative analysis: Fold 1 achieved the highest validation accuracy (89%) among all EfficientNetB0 folds, matching the model’s overall average performance. More importantly, Fold 1 showed exceptional performance for critical conditions - it attained the highest F1-score for Pneumonia (0.79) among all EfficientNetB0 folds and demonstrated strong performance for Cardiomegaly (F1: 0.75, AUC: 0.95). Although Fold 1 showed lower precision for Pneumothorax (0.48), it maintained a clinically favorable high recall (0.83) for this critical condition, minimizing the risk of missed diagnoses. The consistent performance across multiple metrics without significant outliers makes Fold 1 an ideal candidate for comprehensive visualization and analysis.

We recommend presenting Fold 1 of EfficientNetB0 as the primary configuration, with accompanying figures (Loss evolution, Accuracy evolution, Multi-label confusion matrix, ROC curves, and prediction examples) generated from this fold. This selection ensures that readers observe performance characteristics that are both representative of the model’s overall capability and optimized for critical clinical applications.

**Fig. 2:**
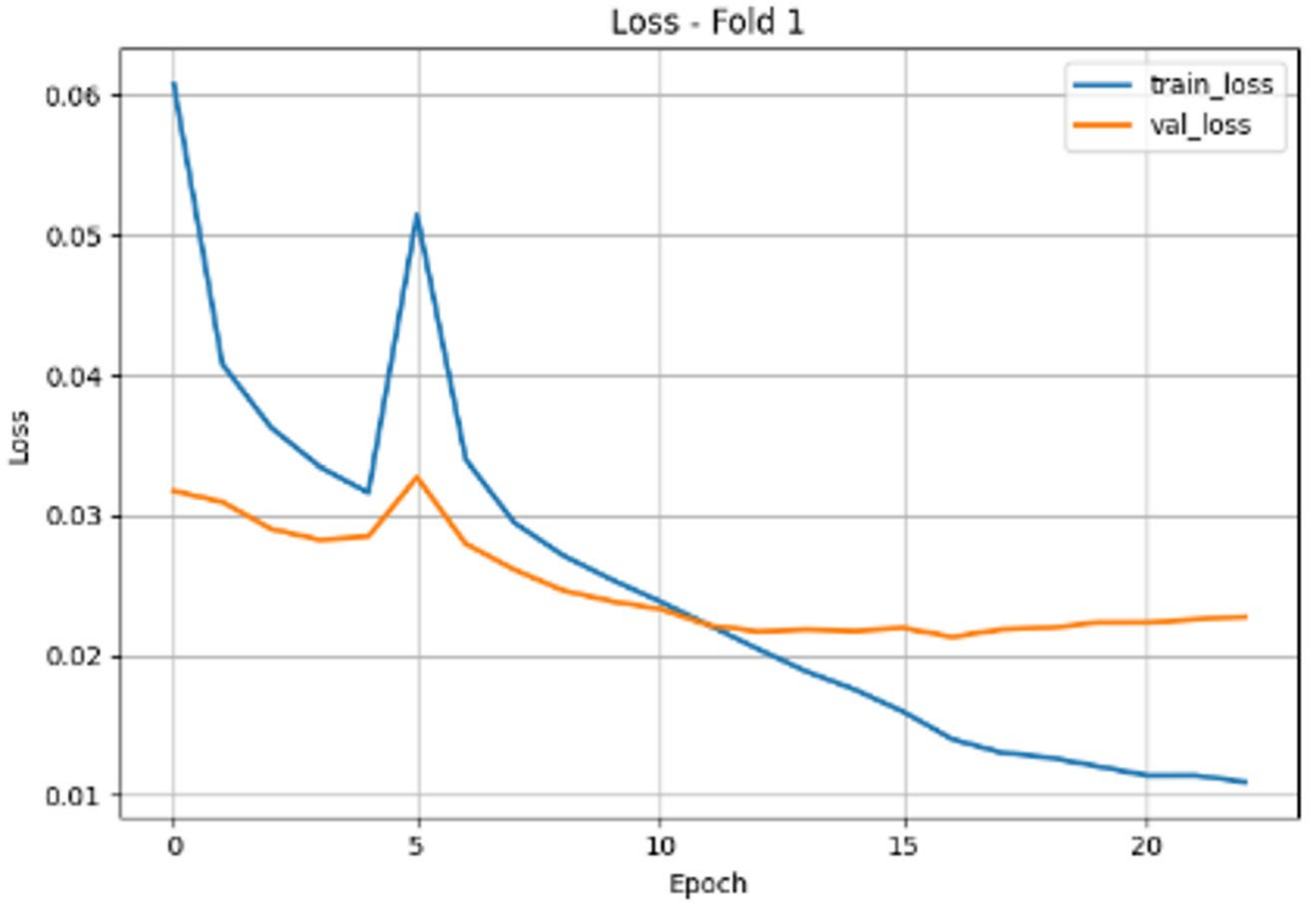
EfficientNetB0(Fold1) loss evolution.

Figure 2 shows the loss evolution plot for EfficientNetB0 (Fold 1) that illustrates the training dynamics over 20 epochs, highlighting both learning progress and emerging overfitting. Training loss (blue) shows a consistent decline from approximately 0.04 at initialization to around 0.02 by epoch 20, indicating effective gradient-based optimization and feature assimilation. In contrast, validation loss (orange) initially decreases in tandem, reaching its minimum of approximately 0.03 at epoch 3. However, beyond this point, validation loss begins to plateau and slightly increase, settling around 0.03–0.035 by epoch 20, while training loss continues to decrease. This subtle but definitive divergence from a near-zero gap at epoch 3 to a gap of approximately 0.01–0.015 by epoch 20 signals the onset of overfitting, where the model becomes increasingly specialized to the training data at the expense of generalization. The optimal stopping point for this fold occurs at epoch 3, where validation loss is minimized; balancing model learning with generalization capability before overfitting begins to degrade performance.

**Fig. 3:**
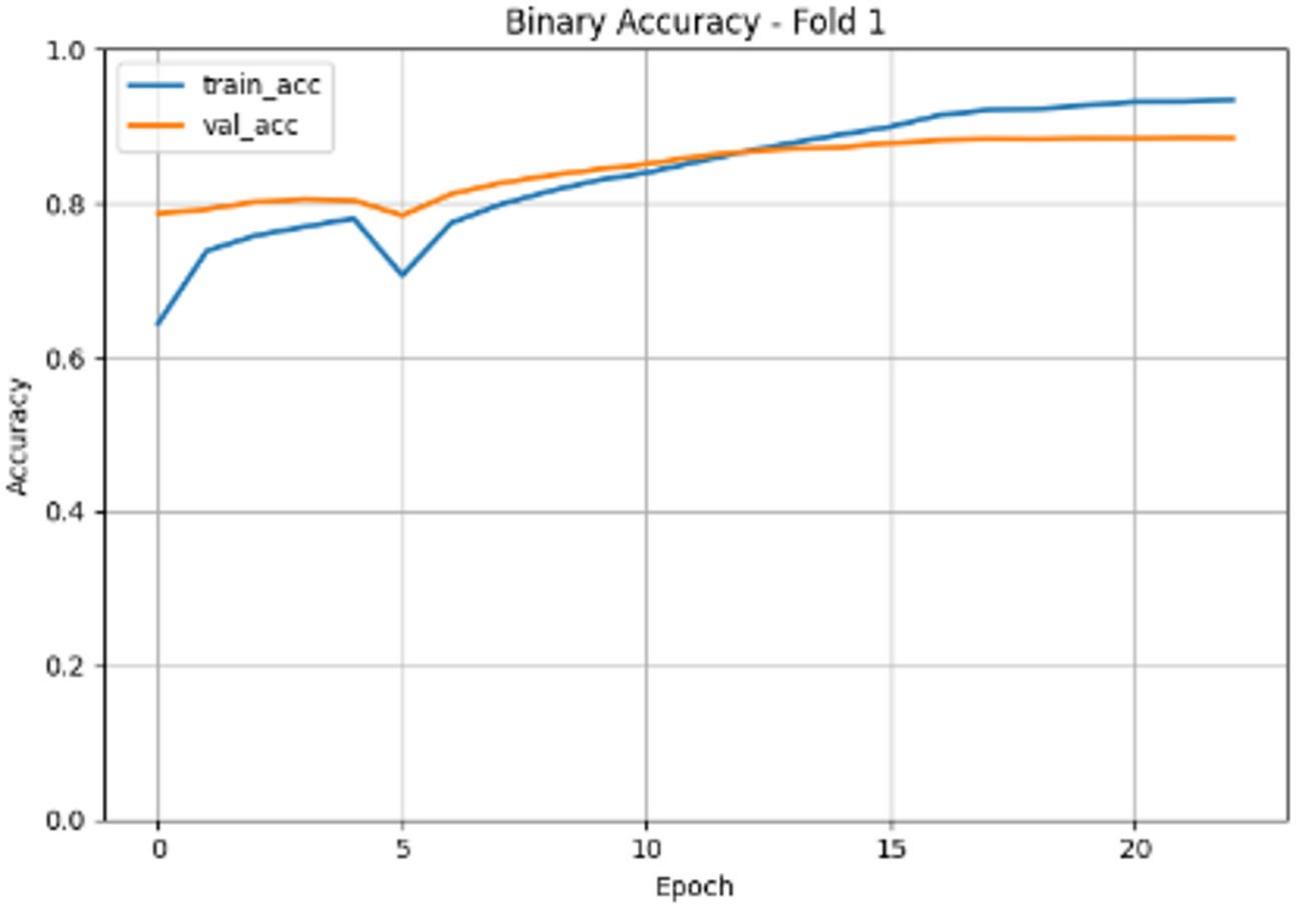
EfficientNetB0(Fold1) accuracy evolution.

Figure 3 shows the accuracy evolution plot that illustrates the learning progress and generalization behavior of the EfficientNetB0 model over 20 epochs. Training accuracy (train_acc) demonstrates robust learning, rising from approximately 0.90 at initialization and approaching near-perfect convergence (~ 0.99) by epoch 20. Validation accuracy (val_acc) follows a distinct trajectory: it increases rapidly from ~ 0.70 to ~ 0.90 within the first 3–4 epochs, after which it plateaus with only minor oscillations, stabilizing around 0.90 for the remainder of training. These results in a progressively widening gap between training and validation accuracy reaching roughly 0.09 by epoch 20 which corroborates the overfitting trend observed in the loss evolution. The early stabilization of validation accuracy, combined with the absence of late-stage decline, indicates that the model achieves its maximal generalizable performance early in training, further justifying the early stopping criterion identified at epoch 3.

**Fig. 4:**
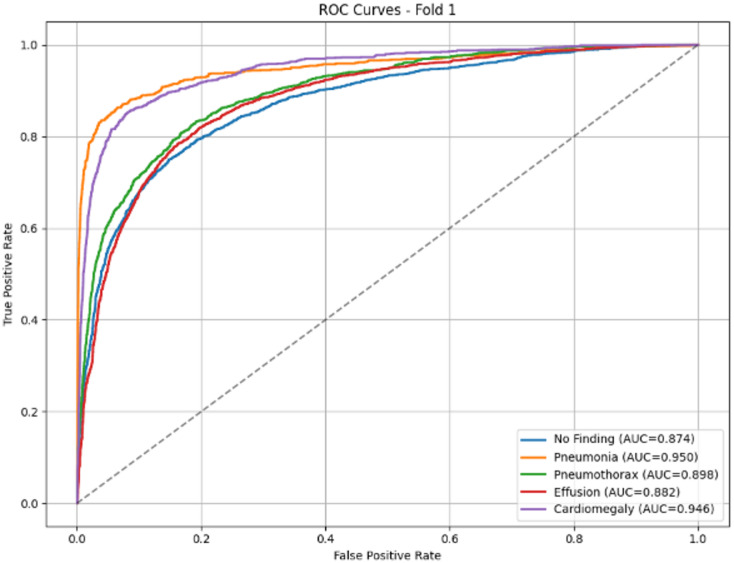
EfficientNetB0(Fold1) ROC-curve.

Figure 4 shows the receiver operating characteristic (ROC) curves that demonstrate the model’s discriminative ability across the five thoracic pathology classes. All classes achieve AUC values above 0.85, confirming strong diagnostic performance. Pneumonia exhibits the highest AUC (0.950), followed closely by Cardiomegaly (0.946), indicating excellent separation of these critical conditions from other classes. Pneumothorax (AUC = 0.898) and Effusion (AUC = 0.882) show robust but slightly lower performance, likely reflecting the subtle and overlapping radiographic presentations of pleural pathologies. The No Finding class (AUC = 0.874), while still demonstrating good discriminative capacity, presents the greatest diagnostic challenge, consistent with the clinical difficulty of confidently classifying normal studies in the presence of potential subtle abnormalities. The overall high AUC values across all categories validate the model’s utility as a screening aid, particularly for high-stakes conditions such as pneumonia and cardiomegaly.

**Fig. 5:**
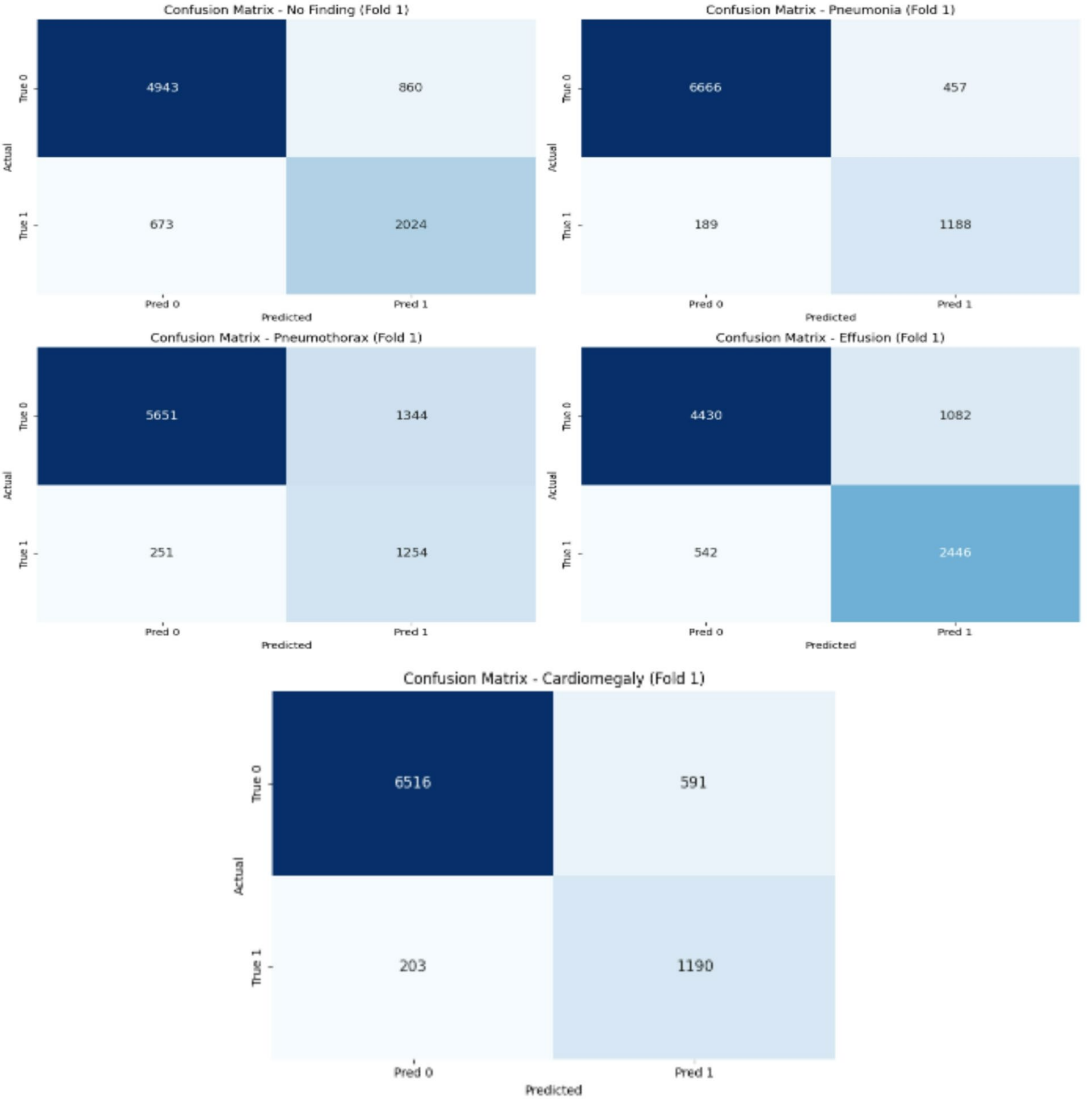
EfficientNetB0(Fold1) confusion matrix.

Figure 5 shows the confusion matrices that reveal distinct diagnostic patterns and challenges across the thoracic pathology classes. For the critical No Finding category, the model demonstrates strong specificity but shows notable false positives, with a portion of normal cases being misclassified as pathological a cautious approach that may enhance sensitivity for disease detection but could increase unnecessary follow-up in clinical settings. Conversely, the Cardiomegaly confusion matrix indicates robust true positive identification but reveals specific misclassification patterns, particularly with conditions exhibiting overlapping cardiac silhouette characteristics. These matrices collectively highlight the model’s strengths in detecting clear pathologies while exposing areas for refinement in distinguishing subtle normal variants from early disease states and resolving inter-class confusion among overlapping thoracic abnormalities.

**Fig. 6:**
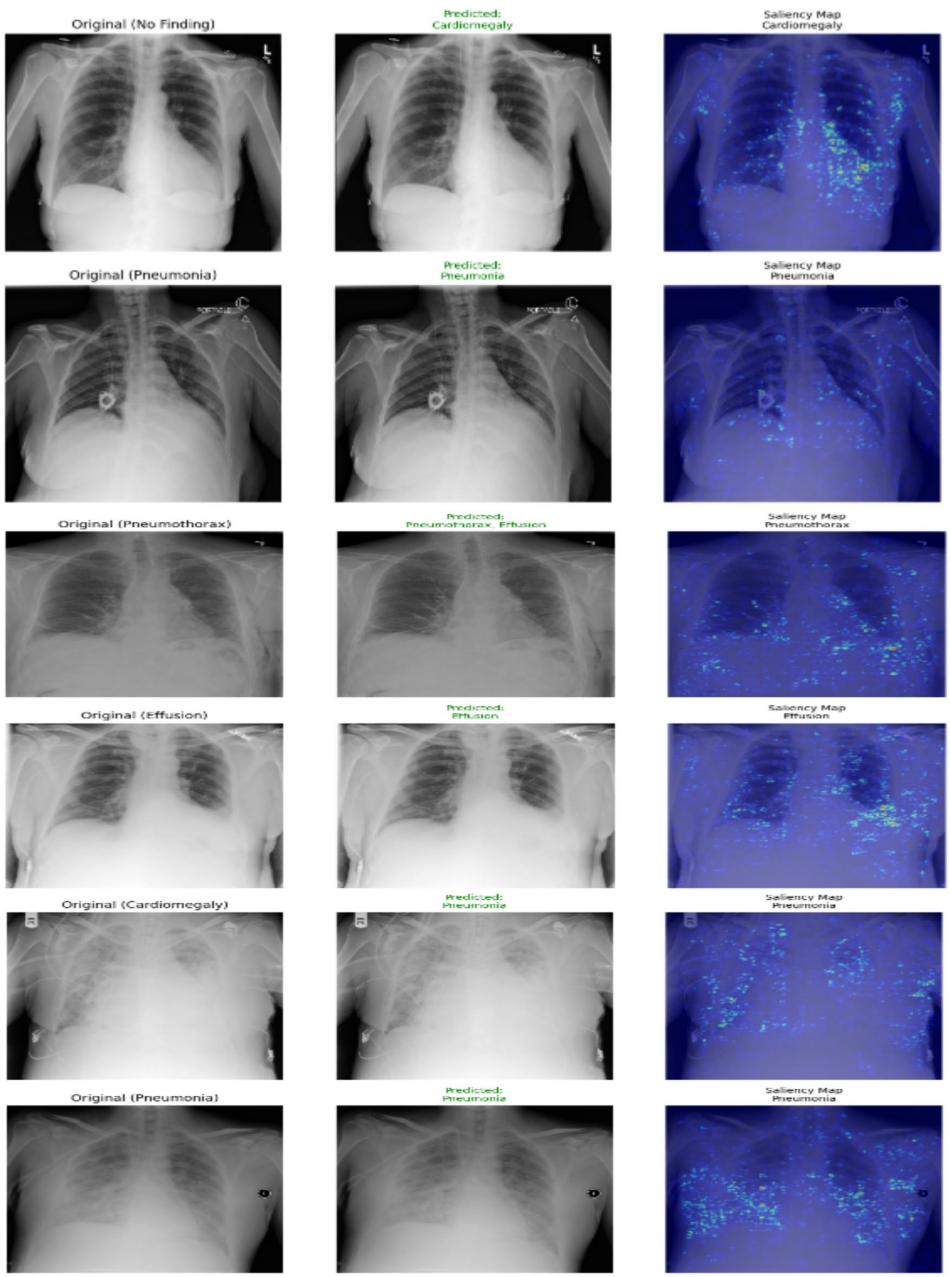
Some predictions using final model.

Figure 6 shows the visualizations with correct and challenging predictions with corresponding saliency maps, providing interpretability into the model’s decision-making process. The saliency maps demonstrate that the model appropriately focuses on clinically relevant regions highlighting pulmonary consolidation areas in correctly classified pneumonia cases, pleural margins in pneumothorax predictions, and cardiac silhouette contours in cardiomegaly assessments. However, the examples also reveal critical error patterns, particularly in cases of effusion-pneumothorax confusion where the model attends to overlapping pleural regions, and in the misclassification of normal studies as pathological where it may be responding to benign anatomical variants or imaging artifacts. These visual explanations not only validate the model’s alignment with radiological reasoning but also pinpoint specific diagnostic ambiguities that mirror real-world clinical challenges, offering transparent insights for both model refinement and potential clinical deployment.

## Conclusion

This study established an optimized multi-label EfficientNetB0 framework for thoracic pathology classification that preserves the clinical reality of co-occurring conditions in chest X-ray analysis. The proposed approach integrates a systematic preprocessing pipeline (CLAHE enhancement with radiological parameter optimization), a per-class balancing strategy that retains multi-label samples, and an enhanced architecture incorporating a Squeeze-Excitation attention block and Focal Loss to address class imbalance and label noise. Through rigorous three-fold cross-validation, the framework demonstrated robust diagnostic performance, achieving a macro-average AUC of 0.906 and superior recall (0.824), with particularly strong discrimination for Pneumonia (AUC = 0.950) and Cardiomegaly (AUC = 0.946). The two-phase transfer-learning strategy (feature extraction followed by fine-tuning) proved effective in adapting EfficientNetB0 to the thoracic imaging domain while maintaining generalization. The results confirm that the framework successfully balances learning capacity and clinical applicability in a realistic multi-label setting a critical advancement over conventional single-label simplifications. While the pipeline has been validated on the NIH dataset, its modular design (preprocessing, balancing, and architecture components) is readily adaptable to other chest X-ray collections and imaging modalities.

## Limitations and future directions

While this study demonstrates an effective multi-label classification framework on the NIH Chest X-ray dataset, several limitations should be acknowledged. First, although we preserved the multi-label nature of the dataset allowing the model to learn from co-occurring pathologies the NIH labels are derived from automated NLP extraction of radiology reports and may contain residual label noise. Rather than discarding potentially valuable multi-label samples, we addressed noise indirectly through robust training techniques (focal loss, augmentation, regularization); however, dedicated noisy-label learning strategies such as co-teaching, label correction, or curriculum learning could further improve robustness and will be explored in future work. Second, validation on a single publicly available dataset limits the assessment of generalizability across different patient populations, imaging protocols, and institutional settings. Third, while the model shows strong performance on the five selected pathologies, extending it to a larger set of thoracic findings (including rare or subtle conditions) would increase clinical utility. Future work will therefore focus on: (1) implementing advanced noisy-label learning techniques to handle label uncertainty more explicitly; (2) multi-dataset and multi-center validation using external CXR collections (e.g., CheXpert, MIMIC-CXR); (3) expanding the classification to include additional pathologies and integrating clinical metadata to enhance context-awareness; and (4) prospective clinical testing to evaluate real-world diagnostic impact and workflow integration.

## Data Availability

The NIH ChestX-ray dataset used in this study is publicly available and de-identified. We accessed the dataset from the official NIH Clinical Center repository (https://nihcc.app.box.com/v/ChestXray-NIHCC; accessed 2026 Feb 24) and used it under its stated data-use and attribution terms, including citation of the original dataset publication^29^. For transparency, we note that a Kaggle mirror exists (https://www.kaggle.com/datasets/nih-chest-xrays/data). The code used for data preprocessing, model training, and evaluation is publicly available via Zenodo at https://doi.org/10.5281/zenodo.18762869.
